# The Mechanism of G Protein-Coupled Receptor Regulation of Ferroptosis in Hepatic Ischemia–Reperfusion Injury

**DOI:** 10.3390/ijms27062866

**Published:** 2026-03-22

**Authors:** Die Hu, Lei Sun, Mei Su, Xuekun Xing

**Affiliations:** 1 School of Public Health, Guilin Medical University, Guilin 541199, China; 25207071007@stu.glmu.edu.cn (D.H.); 25207061018@stu.glmu.edu.cn (L.S.); 19162159634@163.com (M.S.); 2 Guangxi Key Laboratory of Environmental Exposomics and Entire Lifecycle Health, School of Public Health, Guilin Medical University, Guilin 541199, China

**Keywords:** G protein-coupled receptor, hepatic ischemia–reperfusion injury, ferroptosis, lipid peroxidation, iron metabolism

## Abstract

Hepatic ischemia–reperfusion injury (HIRI) is a significant clinical challenge in the field of liver surgery and transplantation, and its pathological mechanisms are complex. In recent years, ferroptosis, a novel form of iron-dependent programmed cell death, plays a central role in this injury process. G protein-coupled receptors (GPCRs), as the largest family of membrane receptors in the body, regulate cellular stress and death through extensive signaling networks. This review elucidates the specific molecular mechanisms by which GPCRs regulate ferroptosis in HIRI by affecting key pathways such as lipid peroxidation, iron metabolism homeostasis, and antioxidant defense. It further explores potential therapeutic strategies targeting specific GPCRs to modulate ferroptosis, thereby alleviating liver injury and improving postoperative outcomes, to provide new insights and a theoretical basis for clinical translation.

## 1. Introduction

Hepatic ischemia–reperfusion injury (HIRI) is a significant cause of hepatic insufficiency associated with clinical procedures such as hepatectomy and liver transplantation. Its complex pathophysiological mechanisms involve the interplay of oxidative stress, inflammatory responses, and various forms of cell death [1]. In recent years, ferroptosis—an iron-dependent form of regulated cell death characterized by the excessive accumulation of lipid peroxides—has been established as a key component in HIRI [2,3]. This process is mechanistically linked to glutathione (GSH) depletion, impaired glutathione peroxidase 4 (GPX4) activity, dysregulated iron metabolism, and extensive lipid peroxidation. Notably, this phenomenon is markedly exacerbated in injuries involving marginal donor livers, particularly those with hepatic steatosis [2,4,5,6,7], underscoring its potential as a promising therapeutic target [8].

As the largest family of membrane receptors responsible for sensing alterations in the intracellular and extracellular milieu, G protein-coupled receptors (GPCRs) act as pivotal regulators of cell fate. Within the complex HIRI microenvironment, diverse signaling molecules can profoundly modulate ferroptosis-associated downstream networks through the activation or inhibition of specific GPCRs [9]. Studies have shown that different GPCRs can play pivotal roles in initiating or inhibiting ferroptosis by regulating core nodes such as the system Xc-/GSH/GPX4 axis, iron metabolism homeostasis, and lipid peroxidation, exhibiting diametrically opposite functions (pro-death or protective) [9,10,11].

Therefore, elucidating the intricate regulatory network governing GPCR-mediated ferroptosis in HIRI not only contributes to a deeper understanding of the complex pathophysiological processes of liver injury but also provides a novel target perspective for developing new and precise liver protection strategies. This article will systematically review this topic, covering molecular mechanisms, experimental progress, and the challenges and prospects of clinical translation.

## 2. Pathophysiological Association Between HIRI and Ferroptosis

### 2.1. Classic Pathological Process and Cell Death Spectrum of HIRI

HIRI is a severe and often unavoidable complication in liver surgery and transplantation, characterized by a complex pathological process involving multiple stages and various cell types [12]. Traditionally, HIRI is divided into two key stages: the ischemic phase and the reperfusion phase. During the ischemic phase, interruption of blood flow leads to disorders of energy metabolism in hepatocytes, ATP depletion, and dysfunction of cell membrane ion pumps, resulting in intracellular acidosis and disruption of calcium ion homeostasis, predisposing the foundation for subsequent injury [13]. Upon entering the reperfusion phase, with the tissue to of oxygen, a large number of reactive oxygen species (ROS) and reactive nitrogen species (RNS) are explosively generated, triggering severe oxidative stress [14]. The core mechanism entails a specific sequence of events: during the ischemic period, the mitochondrial electron transport chain is inhibited, leading to an excessive accumulation of reducing equivalents (such as NADH, ubiquinone) at sites such as complex I and III; during reperfusion, the sudden influx of oxygen allows these excessively reduced carriers to directly transfer electrons to oxygen molecules, generating superoxide anions (O_2_•^−^) and other ROS at the inner mitochondrial membrane, culminating in the “ROS burst” [15]. This process is the primary driver of HIRI. The ROS burst not only directly damages mitochondria, leading to membrane permeability transition and dysfunction and thereby establishing a vicious cycle of ROS production, but also directly initiates membrane lipid peroxidation and activates various pro-inflammatory and pro-death signaling pathways such as nuclear factor-κB (NF-κB) and mitogen-activated protein kinase (MAPK) [15]. Meanwhile, calcium ion overload coincides with inflammatory cell infiltration (such as neutrophils and macrophages), jointly promoting damage to hepatic parenchymal cells [16]. Studies have shown that oxidative stress and the inflammatory response during the reperfusion phase are key processes that exacerbate liver injury, acting synergistically, thereby resulting in hepatocyte dysfunction, microcirculation disorders, and even tissue structural damage [12].

During HIRI, cell death is the primary cause of liver function loss. In addition to traditional apoptosis and necroptosis, recent studies have confirmed that ferroptosis is a crucial form of cell death in HIRI pathogenesis, especially in hepatocytes [17]. Ferroptosis is an iron-dependent form of regulated cell death characterized by lipid peroxidation. Its mechanism involves glutathione depletion, inhibition of GPX4 activity, and excessive accumulation of phospholipids containing polyunsaturated fatty acid [18]. Notably, similar to the spleen and bone marrow, the liver, as the primary site of iron storage in the body, possesses a physiologically high iron load. This renders intracellular iron more susceptible to mobilization and catalyzing lipid peroxidation when subjected to insults such as ischemia–reperfusion. This explains the heightened intrinsic susceptibility of the liver to ferroptosis at the organ level.

In the HIRI model, downregulation of GPX4 expression and elevated levels of lipid peroxidation are observed. Furthermore, administration of ferroptosis inhibitors (such as Ferrostatin-1 or Liproxstatin-1) significantly alleviates liver injury, implicating ferroptosis in the pathogenesis [19,20]. Compared to normal liver, steatotic liver generate more ROS and experience more severe oxidative stress during IRI, making hepatocytes more sensitive to ferroptosis [15]. Therefore, a comprehensive understanding of the spectrum of cell death, including ferroptosis, in HIRI is crucial for developing targeted therapeutic strategies. Notably, under specific conditions, such as in steatotic livers or donation after circulatory death (DCD) grafts, the contribution of ferroptosis may surpass that of other forms of cell death [21].

### 2.2. Ferroptosis as a Core Feature and Driving Factor in HIRI

Ferroptosis in HIRI is characterized by distinct molecular and biochemical features, centered on the collapse of the antioxidant defense system in hepatocytes, the disruption of iron metabolism homeostasis, and the excessive accumulation of lipid peroxides. Firstly, the depletion of glutathione (GSH) and the inactivation of its downstream effector molecule, GPX4, are key initiating events [18]. During HIRI, an acute burst of oxidative stress causes massive GSH consumption, which inactivates GSH-dependent GPX4; this renders the enzyme unable to scavenge phospholipid hydroperoxides (PL-PUFA-OOH), ultimately disrupting membrane integrity [5]. This dysfunction of the GSH-GPX4 axis has been demonstrated in various HIRI models (such as the fatty liver model and the DCD liver transplantation model), where these models exhibit GPX4 downregulation and increased lipid peroxidation markers (such as 4-HNE) [2,22].

Secondly, the pathological accumulation of intracellular free iron serves as the critical catalyst for lipid peroxidation. During the reperfusion period, oxidative stress can increase the “labile iron pool” through mechanisms such as ferritinophagy [20]; concurrently, the upregulation of transferrin receptor 1 (TfR1) expression also promotes iron uptake [23]. Clinical retrospective analyses indicate that iron overload is a risk factor for injury after liver transplantation [24]. Iron ions catalyze the production of hydroxyl radicals via the Fenton reaction, thereby establishing an iron-dependent positive feedback loop of lipid peroxidation, which strongly drives ferroptosis [25]. Finally, lipid peroxidation itself serves as the executor, with both substrate supply and the catalytic process being intensified. Increased expression of long-chain acyl-CoA synthase 4 (ACSL4) promotes the incorporation of polyunsaturated fatty acids into membrane phospholipids, thereby enhancing their susceptibility to peroxidation [2]. The burst of ROS during the reperfusion phase provides ample initiators for the lipid peroxidation chain reaction [3].

Extensive pharmacological studies provide strong support for the aforementioned mechanisms. Ferroptosis inhibitors (Fer-1, Lip-1) and iron chelators (DFO) have been shown to significantly reduce liver injury and lower lipid peroxidation levels in various animal models of HIRI; conversely, studies have demonstrated that a high-iron diet exacerbates the injury [2,24,26]. Taken together, this evidence establishes the potential of targeting the ferroptosis pathway in the treatment of HIRI [8].

However, the interpretation of the aforementioned pharmacological evidence must take into account potential confounding factors and limitations. First, although small-molecule inhibitors such as Fer-1 and Lip-1 are widely employed as selective tools for ferroptosis, their actions are not exclusively specific. For example, some studies have suggested that at high concentrations, Fer-1 may exert a broader antioxidant effect by scavenging free radicals, rather than acting specifically on the GPX4/GSH axis [27,28]; Lip-1 has been found to potentially influence mitochondrial membrane potential and cellular energy metabolism [29]. These off-target effects suggest that the observed hepatoprotective effects may be partially attributable to broad-spectrum inhibition of oxidative stress or other cell death pathways, rather than from the specific blockade of ferroptosis. Furthermore, the current evidence relies heavily on rodent models, whose liver physiology, metabolism, and immune responses differ from those of humans, which limits the direct translatability of these findings to clinical settings. Finally, the severity and mechanisms of injury induced by different HIRI models (such as cold ischemia, warm ischemia, and fatty liver backgrounds) vary, which may influence the contribution of ferroptosis.

In summary, ferroptosis is undoubtedly a critical cell death mechanism in HIRI, and existing pharmacological evidence provides strong, though not conclusive, support for its therapeutic potential. To confirm its central role and facilitate clinical translation, future research must employ more specific genetic tools (such as conditional gene knockout), validate findings in large animal models, and thoroughly elucidate the interactions between ferroptosis and other damage pathways. Such critical insights will aid in the design of more precise and effective clinical intervention strategies.

## 3. The Signal Transduction Pathways of GPCRs and Their Intersection with the Ferroptosis Regulatory Network

### 3.1. From Classic Signaling Pathways to Biased Signaling: The Molecular Basis of GPCRs in Regulating Ferroptosis

Upon ligand activation, GPCRs primarily initiate complex intracellular signaling networks through two types of downstream effectors—heterotrimeric G proteins and β-arrestins—whose combined outputs determine the cell’s fate under stress, including its susceptibility to ferroptosis [30].

Classic G protein signaling rapidly alters intracellular levels of second messengers (such as cAMP, IP3, DAG, and Ca^2+^) by regulating effector molecules including adenylate cyclase and phospholipase C (PLC), thereby exerting widespread effects on metabolism, transcription, and cytoskeletal reorganization [30]. Crucially, the DAG and Ca^2+^ signals generated by Gq protein-activated PLC directly activate protein kinase C (PKC) and calcium-dependent phospholipase A_2_ (PLA_2_). The polyunsaturated fatty acids (PUFAs) released by PLA_2_ serve as key substrates for lipid peroxidation, thereby directly coupling GPCR signaling to the core execution mechanisms of ferroptosis [9].

Traditionally, β-arrestin has been regarded as a protein that mediates receptor desensitization and endocytosis. However, it also serves as an important scaffolding protein capable of independently activating key survival/stress signaling pathways such as MAPK (e.g., ERK, p38, JNK) and AKT [31]. β-arrestin and G protein signaling pathways are not always mutually exclusive; they can operate independently or in concert, forming the basis of “biased signaling” [30]. For example, specific GPCR agonists can preferentially activate β-arrestin or specific G protein subtypes, thereby triggering distinctly different downstream biological effects [32].

This “biased signaling” property offers a revolutionary strategy for precisely targeting ferroptosis. For a given GPCR, the development of a biased agonist capable of selectively activating its anti-ferroptotic pathways—such as activating protective transcription factors via β-arrestin/ERK or enhancing antioxidant defenses via Gs/cAMP—while avoiding the activation of pro-ferroptotic or harmful side-effect pathways, holds promise for highly effective and low-toxicity therapies.

### 3.2. Multidimensional Connections Between GPCR Signaling Networks and Ferroptosis Regulatory Nodes

**Figure 1:**
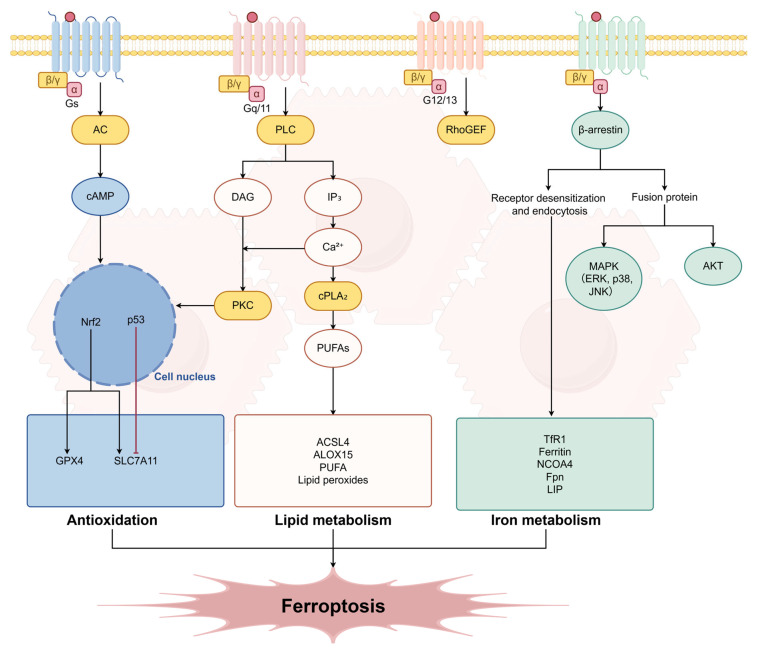
GPCR Signaling Network and Ferroptosis Regulation: A Panoramic View in Hepatocytes.

GPCRs engage in multifaceted crosstalk with the core regulatory nodes of ferroptosis through their downstream signaling networks. First, they regulate transcription via second-messenger coupling. GPCR-activated pathways, such as cAMP/PKA and PKC, can phosphorylate and regulate key transcription factors including Nrf2, p53, and ATF4 [33,34]. For example, GPR68 activation promotes Nrf2 nuclear translocation via the PI3K/Akt pathway, upregulating anti-ferroptosis genes such as SLC7A11 and GPX4 [33]; conversely, signaling from certain GPCRs may inhibit SLC7A11 by activating p53, thereby promoting ferroptosis. Second, they influence iron homeostasis by regulating autophagy. GPCRs can negatively regulate autophagy via the PI3K-AKT-mTOR pathway. When this pathway is inhibited, autophagy (particularly ferritin autophagy) is activated, leading to the release of stored iron and an increase in the labile iron pool, thereby enhancing the cell’s susceptibility to ferroptosis [33]. Furthermore, they interact with organelles through membrane transport. β-arrestin-mediated GPCR endocytosis may influence the degradation efficiency of iron-containing proteins, such as ferritin, through cross-talk with the endosomal-lysosomal system, thereby finely regulating the availability of iron ions [35]. To visually present the multidimensional connections between the GPCRs signaling network and ferroptosis regulatory nodes in hepatocytes, Figure 1 summarizes the macro-architecture of the aforementioned signaling pathways.

### 3.3. The Role of GPCRs in the Regulation of Ferroptosis in Non-Parenchymal Cells

Liver injury results from the interaction between parenchymal and non-parenchymal cells. The signaling activity of GPCRs in Kupffer cells and hepatic stellate cells (HSCs) profoundly influences the ferroptosis process in liver parenchymal cells by modulating the inflammatory microenvironment and intercellular communication.

In Kupffer cells (liver macrophages), the activation of specific GPCRs (such as the peptidoglycan receptor and the complement-opsonized toxin receptor) promotes NLRP3 inflammasome assembly and the release of pro-inflammatory cytokines such as interleukin-1β (IL-1β), exacerbating oxidative stress during the reperfusion phase and thereby indirectly leading to ferroptosis in hepatocytes [36]. Conversely, activation of other GPCRs (such as the prostaglandin E2 receptor EP4) may promote the polarization of Kupffer cells toward an anti-inflammatory phenotype, thereby exerting a protective effect. In HSCs, GPCR activation plays a central role in fibrosis. Multiple GPCRs (such as phosphatidylserine receptors and endothelin receptors) drive the activation and proliferation of HSCs and stimulate the secretion of pro-fibrotic factors (such as TGF-β), which not only alter the extracellular matrix but may also influence the metabolic homeostasis and death thresholds of neighboring hepatocytes through paracrine signaling [37]. Although there are few reports on the direct regulation of ferroptosis by GPCRs within HSCs, activated HSCs are metabolically active, and their lipid metabolism and redox status may be modulated by GPCRs, which in turn may influence lipid peroxidation levels in the local microenvironment.

Therefore, a comprehensive analysis of the differential roles of GPCR signaling within the networks formed by different cell types in the liver is crucial for understanding the global regulatory mechanisms of ferroptosis in HIRI and for designing combination therapeutic strategies targeting specific cell populations.

## 4. The Regulatory Role of Specific GPCRs Family in Ferroptosis During HIRI

### 4.1. The Regulatory Role of Chemokine Receptors (Such as CXCR4, CCR2)

In the complex pathological network of HIRI, chemokine receptors serve as key “signal hubs,” with particular research focus on CXCR4 and CCR2. Studies in non-diabetic rat models indicate that following hepatic ischemia–reperfusion, damaged hepatocytes and sinusoidal endothelial cells rapidly release numerous chemokine ligands, including stromal cell-derived factor-1 (SDF-1/CXCL12) and monocyte chemoattractant protein-1 (MCP-1/CCL2) [38]. These ligands bind to the corresponding receptors CXCR4 and CCR2, which are widely expressed in the liver, initiating a strong chemotactic signal. The core consequence of this process is the recruitment and activation of neutrophils, monocytes/macrophages, and other inflammatory cells, leading to massive infiltration of these cells into the injured liver tissue. Activated inflammatory cells produce a large amount of ROS through respiratory burst and release pro-inflammatory cytokines such as tumor necrosis factor-α (TNF-α) and interleukin-1β (IL-1β), which together exacerbate the oxidative stress and inflammatory cascade in the local tissue [39]. This chemokine receptor-mediated, sustained, and amplified inflammatory microenvironment directly disrupts the redox balance of cells. On the one hand, excessive ROS directly attack polyunsaturated fatty acids on the cell membrane; on the other hand, inflammatory factors can interfere with the iron metabolism homeostasis and the function of antioxidant systems (such as the glutathione system) in cells. Therefore, chemokine receptors, by driving the infiltration of inflammatory cells, effectively create a “fertile soil” rich in oxidative stress and lipid peroxidation substrates for the occurrence of ferroptosis, tightly coupling simple inflammatory damage with ferroptosis.

As a canonical Gi protein-coupled receptor, CXCR4 regulates multiple downstream pathways upon activation, profoundly influencing cell fate decisions. When CXCR4 binds to its ligand CXCL12, the receptor first induces dissociation of the Gi protein into subunits, thereby activating it [40]. The activated Giα subunit directly inhibits adenylate cyclase (AC) activity, leading to decreased intracellular cyclic adenosine monophosphate (cAMP) levels, thereby reducing protein kinase A (PKA) activation [41]. The inhibition of PKA activity may diminish PKA-mediated transmission of certain pro-survival signals. Meanwhile, the released Gβγ dimer can activate key members of the mitogen-activated protein kinase (MAPK) family, including extracellular signal-regulated kinase (ERK) and p38 MAPK. Activation of these two pathways may promote ferroptosis. Studies have shown that sustained ERK activation can upregulate the expression of iron uptake-related proteins such as transferrin receptor 1 (TFR1), increasing the intracellular labile iron pool and providing catalysts for the Fenton reaction. In regulatory T cells, PDK1 upregulates CD71 (also known as TFR1) expression by maintaining the activity of the MEK-ERK signaling pathway; when this pathway is disrupted, TFR1 expression is downregulated [42]. Similarly, in an apolipoprotein E (ApoE)-knockout mouse model, iron accumulation in the liver and spleen was found to be associated with phosphorylated ERK (pERK)-mediated upregulation of TFR1 expression [43]. Furthermore, in retinal pigment epithelial cells, inhibition of ERK phosphorylation leads to reduced TFR1 expression, providing further evidence of the positive regulatory role of ERK activation on TFR1 [44]. On the other hand, the activation status of ERK also directly influences the levels of intracellular labile iron pools. In an ovarian cancer spheroid model, ERK activation promotes protective autophagy; when ERK is blocked using the inhibitor U0126, autophagy is impaired, leading to abnormal accumulation of iron in lysosomes and a reduction in the cytoplasmic labile iron pool. This suggests that sustained ERK activation helps maintain or increase the cytoplasmic iron pool available for reactions [45].The activation of p38 MAPK is closely related to the promotion of lipid peroxidation, which may weaken the cell’s key defense capability to clear lipid peroxides by inhibiting the synthesis or activity of GPX4. Studies have shown that inhibiting the p38 MAPK signaling pathway in models of neuropathic pain significantly reduces levels of malondialdehyde (MDA), a marker of lipid peroxidation [46]. One of the key mechanisms underlying this promotional effect is that p38 MAPK inhibits the synthesis or activity of GPX4, a critical defense molecule involved in the cellular clearance of lipid peroxides. For example, in a glutamate-induced retinal excitotoxicity model, glutamate stimulation upregulates the expression of phosphorylated p38 MAPK (p-p38 MAPK), accompanied by a significant decrease in GPX4 protein levels [47]. However, upon treatment with the p38 MAPK-specific inhibitor SB202190, not only was the activation of p-p38 MAPK inhibited, but GPX4 expression was also restored, thereby suppressing ferroptosis and lipid peroxidation [47]. Similarly, in liver cancer cells, activation of p38 MAPK promotes lipid peroxidation and ferrocytosis, while treatment with the p38 MAPK inhibitor SB203580 partially rescues this effect; its mechanism of action is also related to the regulation of ferrocytosis-associated proteins such as GPX4 [48]. Therefore, the CXCR4-Gi signaling axis reshapes the cell’s metabolism and stress state through a “dual approach”—that is, inhibiting the classical AC-cAMP-PKA survival signal while activating the ERK/p38 MAPK stress signal—making it more prone to ferroptosis.

### 4.2. Purinergic Receptors (Such as P2Y Receptors and Adenosine A2A Receptors) Dual Regulation

Purinergic receptors play a complex and often antagonistic role in HIRI, and their regulatory effects are highly dependent on the receptor subtype and the specific G protein subtypes to which they are coupled. During reperfusion, damaged cells release large quantities of extracellular nucleotides, including ATP and ADP, which act as danger signals to activate specific purinergic receptors [49]. Among them, the P2Y receptor family, comprising G protein-coupled receptors, responds to extracellular ATP, ADP, UTP, and other nucleotides [50]. For example, P2Y1 and P2Y6 receptors are primarily coupled with Gαq/11 proteins, and their activation can lead to mobilization of intracellular calcium ions and protein kinase C (PKC) activation [51]. This signaling cascade has been confirmed in various pathological processes and may exacerbate cell damage by promoting ROS generation and lipid peroxidation [50]. In the context of HIRI, abnormal elevation of extracellular ATP levels is linked to exacerbated damage; furthermore, the absence of NTPDase8 (an enzyme regulating extracellular ATP) worsens liver injury alongside higher ATP concentrations, implicating P2 receptor signaling in the injury process [52]. Therefore, the large release of ATP/ADP during reperfusion may serve as an important pathway for promoting the generation of lipid peroxidation products associated with ferroptosis by activating Gq/11-coupled P2Y receptors.

In contrast to the damaging effects of P2Y receptors, adenosine exerts a clear cytoprotective effect by activating its specific receptors (such as the A2A receptor). Extracellular ATP can be rapidly hydrolyzed to adenosine by ecto-nucleotide triphosphate diphosphohydrolase (such as CD39 and CD73), thereby converting pro-inflammatory purinergic signals into anti-inflammatory and cytoprotective signals [49,53]. Adenosine A2A receptors are coupled with Gs proteins, and their activation can increase intracellular cyclic adenosine monophosphate (cAMP) levels, subsequently activating protein kinase A (PKA) [54]. This pathway has powerful anti-inflammatory and antioxidant effects. Studies have shown that A2A receptor agonists can modulate the expression of thrombospondin-1 (TSP-1) in endothelial cells, indicating their important role in vascular responses and inflammation [55]. Importantly, adenosine receptor signaling is closely related to the core transcription factor of the antioxidant defense system, nuclear factor erythroid 2-related factor 2 (Nrf2). Research has confirmed that A1 and A2B adenosine receptors can inhibit ferroptosis in cardiomyocytes by upregulating the expression of GPX4 [56]. Although this study focused on the myocardium, its mechanism suggests that in the liver, A2A receptor activation may also combat reperfusion-induced oxidative stress and ferroptosis through similar pathways. These pathways enhance Nrf2-mediated antioxidant responses, including upregulating key ferroptosis-related proteins such as GPX4 [57]. In cholangiocyte IRI, A2A and A2B receptor pathways are also believed to inhibit immune-mediated damage and promote cell survival [53].

In summary, the purinergic signaling system exhibits a distinct dual regulatory role in ferroptosis during HIRI. These contrasting effects mainly depend on the differences in receptor subtypes and their downstream signaling pathways: pro-inflammatory P2Y receptors (such as Gq/11-coupled P2Y1 and P2Y6) may promote lipid peroxidation and ferroptosis through calcium signaling and PKC activation; while the anti-inflammatory adenosine A2A receptor (Gs-coupled) enhances the antioxidant capacity of cells via the cAMP/PKA pathway, potentially inhibiting ferroptosis by stabilizing Nrf2 and upregulating GPX4 [51,56]. This balance has been reflected in immune cells; for example, pro-inflammatory M1 and anti-inflammatory M2 macrophages express distinctly different purinergic receptor profiles [51]. In HIRI, the dynamic equilibrium of extracellular ATP/adenosine is precisely regulated by ecto-nucleotide triphosphate diphosphohydrolase, and any disturbance may shift the outcome toward either damage or protection [52,58]. Therefore, targeting specific purinergic receptor subtypes, such as inhibiting pro-damage P2Y signaling or enhancing protective A2A receptor signaling, may provide new therapeutic strategies for preventing and treating HIRI-related ferroptosis.

### 4.3. The Influence of Adrenergic Receptors and Bile Acid Receptors

The pathophysiological process of HIRI involves a complex signaling network, in which adrenergic receptors belonging to the G protein-coupled receptor family and bile acid receptors play key but distinct roles. Depending on the G-protein subtype they are coupled to, adrenergic receptors exhibit opposite effects on ferroptosis in ischemia–reperfusion injury (IRI). The α1-adrenergic receptor primarily couples to the Gq protein, and its activation initiates the phospholipase C (PLC) signaling pathway, leading to increased production of inositol trisphosphate (IP3) and diacylglycerol (DAG). In research models of diabetic retinopathy, it has been demonstrated that under high-glucose conditions, α1-AR activates PLC, which in turn induces calcium ion release via IP3, thereby contributing to the generation of reactive oxygen species [59]. IP3 promotes the release of calcium ions (Ca^2+^) from the endoplasmic reticulum, while DAG activates protein kinase C (PKC). In the context of IRI, excessive activation of this pathway can trigger intense vasoconstriction, exacerbating hepatic microcirculation disorders and further expanding the ischemic area. Critically, the resulting sustained increase in intracellular calcium ion concentration (calcium overload) can disrupt mitochondrial function and promote the excessive production of ROS. Calcium overload can also activate calcium-dependent proteases, such as calpain, which degrade the cytoskeleton and compromise the integrity of cell membranes. These events collectively create a pro-oxidative and pro-lipid peroxidation microenvironment, indirectly but significantly promoting the occurrence of ferroptosis. The hallmark of ferroptosis is iron-dependent lipid peroxidation. Therefore, the signaling of α1-adrenergic receptors may serve as a bridge connecting hemodynamic disturbances and cellular ferroptosis in IRI.

In contrast to the deleterious effect of α1-adrenergic receptors, β2-adrenergic receptors, as Gs protein-coupled receptors, are typically associated with cytoprotective effects upon activation. When β2-adrenergic receptors are activated by their ligands (such as adrenaline), they stimulate adenylate cyclase (AC), leading to an increase in intracellular cyclic adenosine monophosphate (cAMP) levels. cAMP, acting as a second messenger, cAMP primarily activates protein kinase A (PKA) its main downstream effector [60]. The activated cAMP-PKA signaling axis mediates multiple protective effects. Firstly, PKA can phosphorylate and inhibit pro-apoptotic proteins such as BAD. Additionally, it activates transcription factors such as CREB to promote the expression of anti-apoptotic genes. Studies have shown that when PKA activity is inhibited (e.g., by treatment with carbamazepine), the level of BAD phosphorylation in platelets decreases, which promotes apoptosis [61]. This pathway may also counteract oxidative stress by regulating energy metabolism, improving mitochondrial function, and enhancing cellular antioxidant defense capabilities. In the case of ferroptosis, although more evidence is needed to establish a direct link between β2-adrenergic receptor signaling and the classical ferroptosis pathway (such as the SLC7A11-GPX4 axis), it is likely that this signaling indirectly suppresses ferroptosis. By alleviating overall oxidative damage and maintaining cellular energy homeostasis, it reduces the triggering conditions for ferroptosis, thereby providing protection in ischemia–reperfusion injury (IRI).

The bile acid receptor TGR5 (also known as GPBAR1) is another important Gs protein-coupled receptor, and its mechanisms in liver protection, especially in combating cholestasis-related damage and ferroptosis, have been increarsingly elucidated. Upon activation by bile acids, such as ursodeoxycholic acid, TGR5 similarly activates adenylate cyclase through Gs proteins, leading to an increase in cAMP levels and activation of PKA. Studies have shown that TGR5 agonists (such as INT-777) inhibit ferroptosis by activating the TGR5/cAMP/PKA/Nrf2 signaling pathway [62]. Specifically, in a model of renal ischemia–reperfusion injury, treatment with the TGR5 agonist INT-777 activates the downstream cAMP/PKA pathway, thereby promoting the activation of nuclear factor E2-related factor 2 (Nrf2). Activation of this pathway ultimately inhibits oxidative stress and ferroptosis. Consequently, this manifests as reduced intracellular levels of ferrous ions (Fe^2+^), decreased production of reactive oxygen species (ROS), and lower levels of malondialdehyde (MDA), a lipid peroxidation end product [62]. To systematically compare the distinct regulatory mechanisms of different GPCR families in ferroptosis during HIRI, the key features of these receptors—including their ligands, coupled G proteins, downstream signaling pathways, effects on ferroptosis, and corresponding evidence—are summarized in Table 1.

## 5. The Key Downstream Molecular Mechanisms of GPCRs Regulating Ferroptosis

### 5.1. The Impact on the System Xc-GSH-GPX4 Antioxidant Axis

GPCR signaling bidirectionally regulates the Xc-GSH-GPX4 axis, which is critical for determining cellular resistance to ferroptosis. In terms of protective signaling, activators of G protein-coupled receptor 68 (GPR68) can promote the nuclear translocation of nuclear factor E2-related factor 2 (Nrf2) via the PI3K/Akt pathway. Nrf2 subsequently upregulates the transcription of solute carrier family 7 member 11 (SLC7A11) and GPX4, enhancing cysteine uptake and GSH synthesis, thereby strengthening antioxidant defense [33]. Conversely, pro-ferroptotic signaling is mediated by specific GPCRs; for example, upregulation of GPR116 inhibits this pathway [11]. The mechanism may involve Gq protein-activated protein kinase C (PKC) or specific MAPK pathways, which in turn activate p53 or activating transcription factor 4 (ATF4). p53 can directly inhibit SLC7A11 transcription, leading to GSH depletion and GPX4 inactivation, ultimately inducing ferroptosis [63,64]. Therefore, GPCRs precisely regulate the activity of the antioxidant axis by influencing key transcription factors such as Nrf2 and p53.

### 5.2. Regulation of Intracellular Iron Metabolism Homeostasis

GPCRs can profoundly regulate intracellular iron homeostasis by influencing iron uptake, storage, and efflux proteins, thereby altering sensitivity to ferroptosis. On the one hand, signaling through specific GPCRs may upregulate transferrin receptor 1 (TfR1), promoting iron uptake [65]. On the other hand, they may influence the release of iron stores; for example, studies suggest that inhibition of the CXC chemokine receptor 3 (CXCR3) can trigger NCOA4-mediated ferritin autophagy, thereby increasing the labile iron pool [66]. In addition, downstream kinases of GPCRs (such as MAPK and PI3K-AKT) may influence the stability of the iron transporter (Fpn) through post-translational modifications, thereby regulating iron efflux [67,68]. The β-arrestin-mediated endocytosis of GPCRs may also indirectly influence the degradation efficiency of iron-containing organelles, such as ferritin, through crosstalk with the endosomal-lysosomal system, thereby finely regulating the release and availability of iron ions [35].

### 5.3. Direct Effects of Lipid Metabolism and Peroxidation

**Figure 2:**
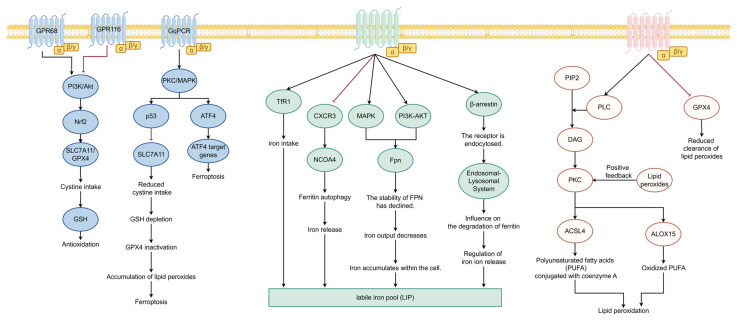
Molecular Mechanisms of GPCRs in Regulating Ferroptosis: A Three-Channel Decomposition.

GPCRs can directly modulate the catalytic and amplification stages of lipid peroxidation. Gq protein-coupled receptor activation triggers phospholipase C (PLC) to produce DAG, which in turn activates PKC. Studies have shown that PKC (including PKCβII) can phosphorylate and activate lipoxygenases (including ALOX15) and long-chain acyl-CoA synthase 4 (ACSL4), thereby directly catalyzing the oxidation of polyunsaturated fatty acids in membrane phospholipids. This process forms a lipid peroxidation-PKCβII-ACSL4 positive feedback axis, thereby potently promoting ferroptosis [69]. In addition, GPCRs can reshape the lipid metabolic environment at the transcriptional level. For example, activation of the G protein-coupled estrogen receptor (GPER) upregulates the expression of ACSL4 and ALOX15 while downregulating GPX4, making the cell membrane more susceptible to oxidation [10]. Similarly, the lipid peroxidation product 4-HNE can trigger downstream cell death signals by activating GPR120 [70]. Together, these mechanisms indicate that GPCRs create the necessary conditions for ferroptosis to occur in HIRI by directly regulating key enzymes involved in lipid peroxidation and lipid composition. Based on the above molecular mechanisms, Figure 2 summarizes the key downstream pathways through which GPCRs regulate ferroptosis in a three-channel format.

## 6. Experimental Research Progress on Targeting GPCRs to Intervene in Ferroptosis in HIRI

### 6.1. Verification of the Liver Protective Effects of GPCR Agonists/Antagonists and Considerations for Translational Applications

In animal models of HIRI, agonists or antagonists targeting specific GPCRs effectively alleviate liver damage, with some protective effects closely related to the inhibition of ferroptosis. This section first elucidates the core mechanisms through which GPCRs regulate ferroptosis and subsequently evaluate their translational potential.

#### 6.1.1. Direct Evidence and Mechanistic Framework for GPCR Regulation of Ferroptosis

Determining whether and how GPCRs directly regulate ferroptosis is central to understanding their protective effects. Current research reveals that GPCRs modulate ferroptosis through multi-layered mechanisms:

Direct Regulation of Key Substrates and Pathways in Ferroptosis: The most direct evidence comes from the adhesion GPCR member GPR56. Its activation directly reduces the abundance of PUFAs on the cell membrane available for lipid peroxidation by promoting the endo-lysosomal degradation of the fatty acid transporter CD36, thereby inhibiting ferroptosis at its source [9].

Direct Regulation of the Core Antioxidant Defense System: Agonists of the bile acid receptor TGR5 can activate pathways such as cAMP-PKA, enhancing the nuclear translocation and transcriptional activity of the transcription factor Nrf2. Although Nrf2 can be directly activated by Keap1 thiol oxidation during ischemia–reperfusion injury (IRI), TGR5 signaling provides crucial synergistic amplification, thereby significantly upregulating the expression of key antioxidant proteins such as GPX4 and heme oxygenase-1 (HO-1) [71], directly countering lipid peroxidation.

Acting as Upstream Signal Sensors and Integrators: The precise initiating signaling pathways for ferroptosis remain a key focus in the field. As the primary signal transducers on the cell surface, GPCRs are poised to act as “initiating sensors” that detect early signals, such as ROS and damage-associated molecular patterns (DAMPs) generated in HIRI. By activating downstream pathways such as the mitogen-activated protein kinase (MAPK) and phosphatidylinositol 3-kinase/protein kinase B (PI3K/AKT) pathways, or by modulating calcium signaling, they subsequently influence lipid metabolism and iron homeostasis, thereby exerting regulatory control at the very upstream of the ferroptosis cascade.

Furthermore, GPCRs can also contribute to the anti-ferroptotic network through indirect mechanisms, including antagonizing chemokine receptors (e.g., CCR4) to suppress inflammatory amplification signals [72], or modulating adenosine receptors to maintain calcium homeostasis.

#### 6.1.2. Validation of Existing Pharmacological Tools and Bottlenecks in Human Translation

Based on the mechanisms described above, various GPCR modulators have shown potential in animal models. Examples include the GPR56 agonist 17-OH PREG, the TGR5 agonist INT-777, and highly selective A1R allosteric modulators (PAMs) [9,71,73]. However, this evidence primarily originates from rodent models, limiting its relevance for clinical translation.

To overcome this bottleneck, future research must focus on human-centric validation strategies: ① Utilize human liver organoids/primary hepatocytes to validate the anti-ferroptosis effects and mechanistic conservation of the aforementioned modulators in human cells; ② Analyze clinical cohort samples to establish correlations among the expression of specific GPCRs (such as TGR5, GPR56), ferroptosis markers (4-HNE, PTGS2), and clinical outcomes (such as graft dysfunction) to provide a basis for target screening; and ③ Explore drug repurposing to evaluate the potential of GPCR-related drugs already in clinical stages (such as bile acid preparations, adenosine receptor modulators) for treating HIRI.

### 6.2. Exploration of Multi-Target Synergistic Intervention Strategies

Given the complexity of the HIRI mechanism, combined intervention strategies hold greater promise. Their design should be based on an understanding of GPCR mechanisms and always be guided by clinical translation.

#### 6.2.1. Design of Synergistic Strategies Based on Mechanism Complementarity

Cross-level combination (intervention initiation and execution): Combining GPCR modulators (e.g., GPR56 agonists) to intervene in ferroptosis initiation signals or substrate supply, and using them in combination with downstream execution inhibitors such as Ferrostatin-1 to achieve full pathway coverage.

Spatiotemporal sequential and cell-specific combination: Based on the evolution of cell death modes during the HIRI process [74], designing sequential regimens (e.g., using ferroptosis inhibitors to protect hepatocytes before reperfusion, and later combining them with GPCR modulators that regulate Kupffer cells). Utilizing targeted nano-delivery systems [75] enables precise drug delivery to specific cell populations.

#### 6.2.2. Strategy Evolution Path Centered on Clinical Translation

The exploration of all synergistic strategies must be closely integrated with human data to form a closed translational loop: ① First, validate the synergistic efficacy in human-derived organoid models; ② Utilize clinical biomarkers and patient stratification (e.g., combined fatty liver disease, diabetes) to guide the development of personalized combination regimens for different subgroups; and ③ Prioritize exploring the combination potential of GPCR-targeted drugs that are already approved or in clinical development to accelerate the clinical translation process.

## 7. Challenges and Future Research Directions in Clinical Translation

### 7.1. Tissue Specificity and Off-Target Effects

GPCRs are widely expressed in various tissues and organs throughout the body. This characteristic raises significant concerns about off-target effects and the risk of extrahepatic side effects when systemic drug therapies targeting GPCRs are employed to modulate HIRI related ferroptosis. For example, in cancer treatment, off-target toxicity caused by the non-specific delivery of conventional chemotherapy drugs significantly limits their efficacy [76]. This challenge is equally prominent in GPCR-targeted therapies for HIRI, as many GPCR subtypes also exert critical physiological functions in critical organs such as the cardiovascular system and central nervous system. Systemic activation or inhibition of these receptors may precipitate severe adverse reactions. Therefore, developing liver-specific targeted delivery systems or utilizing prodrug strategies to achieve specific release and activation of drugs at liver lesion sites has become an important research direction in this field [77].

Smart delivery technologies designed to overcome the challenges of liver targeting have already demonstrated proof-of-concept success in other liver disease models, providing a feasible reference for their application in HIRI. For example, nanoparticles designed to deliver ROS-responsive prodrugs or drugs can utilize the intense oxidative stress that erupts during the reperfusion phase of HIRI as a trigger signal to release the drug specifically in the damaged liver, thereby minimizing exposure to normal tissues [77,78]. Similarly, nanocarriers modified with hyaluronic acid or galactose can facilitate drug accumulation in specific liver cell types by targeting corresponding receptors on the surface of hepatic stellate cells or hepatocytes, such as CD44 or the desialylated glycoprotein receptor [79]. When combined with GPCR modulators, these technologies are expected to significantly enhance therapeutic precision and safety.

For instance, some studies have designed pH-sensitive nanoparticles that are “activated” only under the weakly acidic conditions of the tumor microenvironment, effectively avoiding off-target toxicity in normal tissues [77]. Similarly, designing responsive nano-delivery platforms targeting the HIRI microenvironment (such as hypoxia and acidity) to precisely deliver GPCR modulators to the damaged liver is a feasible path for improving treatment safety and efficacy [76]. Additionally, the use of prodrugs or probes activated by liver-specific enzymes (such as nitroreductase) also represents a strategy for achieving targeted therapy, which has been validated in other disease models [80].

In addition to the tissue-specific challenges in drug delivery, the functional heterogeneity of GPCRs in different liver cell types presents an additional substantial barrier to precision therapy. The liver is a complex organ composed of various cell types, including hepatocytes, Kupffer cells (liver macrophages), and hepatic stellate cells. The same GPCR may mediate distinct or even opposite signaling pathways and biological effects across distinct cell types. Therefore, precisely elucidating the specific functions of certain GPCRs within different cell subpopulations in HIRI is a prerequisite for developing cell type-selective therapies. Addressing this necessitates cell-specific knockout animal models, single-cell sequencing, and advanced imaging technologies (e.g., dual-mode fluorescence/magnetic resonance imaging capable of cell-types discrimination) [81]. Only by defining the cell specificity of the GPCR signaling network can more precise intervention strategies be designed, such as developing drug carriers or modulatory approaches that specifically target hepatocytes or certain non-parenchymal cells. This approach inhibits harmful ferroptosis while avoiding interference with the normal physiological functions of other cells or triggering unnecessary side effects [82].

### 7.2. Complexity of Signaling Pathways and Compensatory Mechanisms

The regulatory network governing ferroptosis in HIRI is highly complex; GPCRs represent only one component of this network, and their downstream signaling pathways involve extensive cross-talk and feedback regulation. For example, it has been demonstrated that the μ-opioid receptor can mitigate ferroptosis in HIRI via the HIF-1α/KCNQ1OT1 axis [83]. However, this protective effect may be disrupted or offset by other parallel pathways. Studies have shown that in ischemia–reperfusion injury of the fatty liver, the roles of lipid peroxidation and ferroptosis are significantly amplified [2]. It is suggested that the predominant ferroptosis-inducing pathways may vary depending on the pathological context. Furthermore, EMP1 in hepatic sinusoidal endothelial cells can drive ferroptosis via the p38 MAPK signaling pathway, thereby exacerbating liver injury [84]. This reveals the critical role of non-parenchymal cells in the ferroptosis network, whose signaling may interact with GPCR signaling within hepatocytes. This cell-type specificity and pathway crosstalk imply that intervention strategies targeting a single GPCR may have their effects attenuated by compensatory mechanisms active in other cell types. For instance, while inhibition of IDO-1 in macrophages can attenuate macrophage-triggered hepatocyte ferroptosis [85], the intrinsic ferroptosis program in hepatocytes may still be activated via other receptors, such as CD36 [86]. Therefore, it is essential to understand the role of GPCRs at the level of the organ’s overall signaling network and to design combined intervention strategies that simultaneously modulate multiple key nodes, in order to avoid treatment failure caused by compensatory mechanisms. Such combination strategies should be cell-type-specific; for example, this could involve the combined use of drugs that protect hepatocytes from ferroptosis alongside agents that regulate the activation status of Kupffer cells or hepatic stellate cells, thereby synergistically blocking the damage cascade at the multicellular level. Furthermore, the pathological process of HIRI exhibits clear sex dimorphism, suggesting that the regulation of ferroptosis by GPCRs may also exhibit sex differences; yet, research in this area remains limited, and conclusions should be interpreted with caution. Studies have found that the DTL-PROX1 signaling axis is a key determinant of gender dimorphism in HIRI-induced injury [87]. Given that the activity or expression of numerous GPCRs (such as the estrogen receptor GPR1/GPR30 and the β2-adrenergic receptor) is regulated by sex hormones, they may play a significant role in mediating sex differences in liver injury and protection. For example, estrogen receptor signaling has been shown to exert antioxidant and anti-ferroptotic effects in various models [88,89]. This may partly explain why female animals exhibit milder damage in certain IRI models. However, this protective effect may vary significantly or even be reversed depending on age, hormone levels, and pathological background (such as postmenopausal status or fatty liver). Therefore, future studies must systematically compare the expression and activation status of key GPCRs, as well as the efficacy of their downstream anti-ferroptotic pathways, across animal models of different sexes. Failure to account for these sex differences may result in GPCR-targeting strategies that are effective in single-sex (typically male) models exhibiting inconsistent efficacy or population-specific biases when translated to the clinical setting.

Ferroptosis itself is finely regulated by various induction and inhibition pathways, and GPCRs may represent only one component in this complex regulatory network, whose relative importance needs to be carefully evaluated in different IRI models and disease stages. In HIRI, multiple ferroptosis regulatory axes independent of classical GPCRs have been identified. For example, SIRT4 can inhibit ferroptosis by deacetylating PRDX3 [90]; Insulin-induced gene 2 (INSIG2) modulates ferroptosis in fatty liver by regulating GPX4 [6]; and the DTL-PROX1 signaling axis is a key determinant of sexual dimorphism in HIRI [87]. These findings emphasize the diversity of ferroptosis regulatory mechanisms. The intervention effects of GPCRs may strongly depend on the specific pathological environment. In a liver transplantation model after cardiac death, the Chinese herbal monomer baicalin exerts a protective effect by regulating ALOX15/iNOS-mediated ferroptosis [22], which is another pathway distinct from typical GPCR pathways. Even within GPCRs, different receptors may produce completely opposite effects. Moreover, the role of ferroptosis varies among different cell types. For instance, in cholangiocyte ischemia–reperfusion injury, ferroptosis inhibitors can protect cholangiocytes [20]. In fatty HIRI-related acute kidney injury, inhibition of liver ferroptosis can indirectly protect the kidneys, although the kidneys themselves do not exhibit significant ferroptosis [91]. This further illustrates that the regulation of ferroptosis by GPCRs may have organ and cell specificity. Therefore, when assessing the importance of GPCRs in HIRI ferroptosis, it is essential to consider model differences (such as normal liver vs. fatty liver, warm ischemia vs. cold ischemia), injury stages (early vs. late), sex differences, and the main types of injured cells (hepatocytes, endothelial cells, or cholangiocytes). Future research needs to systematically compare the relative contributions of GPCR signaling and other ferroptosis pathways (such as the Nrf2/HO-1/GPX4 axis [92], NRF2 pathway [93], and mitophagy [94]) in these different contexts to identify the most therapeutically promising targets. In particular, the precise manipulation of signaling pathways using biased agonists, along with elucidating the functions of GPCRs in non-parenchymal cell networks, will be key breakthroughs in overcoming complexity and compensatory mechanisms to achieve effective combination therapies.

## 8. Conclusions

GPCRs play an important role in the ferroptosis regulatory network in HIRI. Different GPCR subtypes precisely regulate intracellular iron homeostasis, the glutathione antioxidant system, and the lipid peroxidation process, profoundly affecting the execution of ferroptosis. The GPCRs-ferroptosis axis is not an isolated linear pathway but a dynamic network that integrates metabolism, oxidative stress, and cell death signals. Therefore, interventions targeting a single node may yield limited efficacy or incur off-target effects. Future management strategies should focus on “network regulation” and develop multi-target modulators to synergistically stabilize iron metabolism and the antioxidant system.

## Figures and Tables

**Table 1:** The regulatory role of GPCRs families in HIRI on ferroptosis.

GPCR Family	Receptor Subtype	Primary Ligands	Coupled G Protein	Major Downstream Signaling Pathways	Effect on Ferroptosis	Core Mechanisms and Actions	Evidence Level
Chemokine Receptors	CXCR4	CXCL12 (SDF-1) [38]	Gi protein [40]	1. Inhibiting AC → ↓cAMP → ↓PKA [41] 2. Gβγ activating MAPK (ERK/p38 MAPK)	Promote	Dual approach to drive ferroptosis	In vivo [38,39,43,46]; In vitro [40,41,42,44,45,47,48]
	CCR2	CCL2 (MCP-1) [38]	Gi protein (speculated)	Chemotactic signals	Promote	Infiltration of inflammatory cells (monocytes/macrophages).	In vivo [38,39]
Purinergic Receptors	P2Y1, P2Y6 (Pro-injury)	Extracellular ATP, ADP, UTP [49,50]	Gq/11 protein [51]	PLC → IP3/DAG → ↑Ca^2+^, PKC activation [51]	Promote	Exacerbating oxidative damage	In vivo [52]; In vitro [50,51]
	A2A Receptor (Protective)	Adenosine (from ATP hydrolysis via CD39/CD73) [49,53]	Gs protein [54]	AC → ↑cAMP → PKA activation [54]	Inhibit	Enhancing antioxidant defense	In vivo [53,55,56,57]; In vitro [49,53]
Adrenergic Receptors	α1-AR	Epinephrine, Norepinephrine	Gq protein [59]	PLC → IP3/DAG → ↑Ca^2+^, PKC activation [59]	Promote (indirect)	Exacerbating microenvironment and inducing calcium overload	In vivo/In vitro ([59]—diabetic retinopathy model)
	β2-AR	Epinephrine, Norepinephrine	Gs protein [60]	AC → ↑cAMP → PKA activation [60]	Inhibit (speculated)	Maintaining cellular homeostasis	In vitro [60,61]
Bile Acid Receptor	TGR5 (GPBAR1)	Bile acids (e.g., Ursodeoxycholic acid) [62]	Gs protein [62]	AC → ↑cAMP → PKA → CREB activation [62]	Inhibit	Directly blocking ferroptosis execution	In vivo ([62]—renal IRI model)

## Data Availability

No new data were created or analyzed in this study.
